# Application of next-generation sequencing on diagnosis of bloodstream infection caused by *Mycoplasma hominis* in a patient with ANCA-associated vasculitis

**DOI:** 10.1186/s12941-023-00580-4

**Published:** 2023-04-21

**Authors:** Yili Chen, Hengxin Chen, Hao Huang, Yinyin Zhong, Xiaoshu Lin, Peisong Chen, Kang Liao, Juhua Yang, Min Liu, Mengzhi Hong, Ruizhi Wang

**Affiliations:** 1 Department of Laboratory Medicine, The First Affiliated Hospital of Sun Yat-sen University, Guangzhou, Guangdong China; 2 KingMed School of Laboratory Medicine, Guangzhou Medical University, Guangzhou, Guangdong China; 3 Vision Medicals Co., Ltd, Guangzhou, China

**Keywords:** *Mycoplasma hominis*, Bloodstream infection, Next generation sequencing, Diagnosis

## Abstract

**Background:**

*Mycoplasma hominis* is one of the main opportunistic pathogenic mycoplasmas in humans which has a major impact on patients with bloodstream infections. Because it is difficult to detect or isolate, rapid and accurate diagnosis using improved methods is essential and still challenging for patients with bloodstream infection.

**Case presentation:**

In this case, we reported the application of next -generation sequencing for the diagnosis of bloodstream infection caused by *Mycoplasma hominis* in a patient with Antineutrophil cytoplasmic antibody (ANCA)-associated vasculitis. After 9 days of combined treatment with levofloxacin, polymyxin B and meropenem, the patient’s condition was gradually controlled and he was discharged without further complications. During the three-month outpatient follow-up, no recurrence of symptoms or clinical signs was reported.

**Conclusions:**

This successful application of next generation sequencing assisted the rapid diagnosis of *Mycoplasma hominis* bloodstream infection, provided a new perspective in the clinical approach and highlighted the potential of this technique in rapid etiological diagnosis.

## Background

*Mycoplasma hominis* (*M. hominis*) is one of the main opportunistic mycoplasmas in humans capable of causing disease. *M. hominis* mainly resides in the genitourinary tract and sometimes causes upper genitourinary tract infection [1, 2]. It can also cause epididymitis, cervicitis, pelvic inflammatory disease and puerperal fever. However, *M. hominis* is rarely detected in bloodstream infections or isolated from blood cultures [3]. The growth of the mycoplasma requires special growth medium, and the lack of cell wall makes it difficult to culture and identify by ordinary methods. Next-generation sequencing (NGS) is a powerful new technology to identify pathogens which were difficult by traditional methods. Here, we reported a case of *M. hominis* bloodstream infection in an ANCA-associated vasculitis patient with the help of NGS making a rapid diagnosis of *M. hominis* infection. Following the use of NGS, we extended the incubation time for the strain, successfully isolating the bacteria from anaerobic blood culture bottle, and further verified *Mycoplasma homini*s by of MALDI-TOF MS. Finally, the patient was treated successfully with a combination of levofloxacin, polymyxin B and meropenem.

### Case presentation

A 63-year old male was admitted to the hospital (the First Affiliated Hospital of Sun Yat sen University, Guangzhou, China) due to facial edema for 4 months and oral and nasal bleeding for 2 days. Laboratory examinations showed that creatinine 590 µmol/L (normal range 44–133 umol/L), autoantibody the neutrophil proteins leukocyte proteinase 3 (PR3-ANCA) positive, myeloperoxidase (MPO) positive, antinuclear antibody 1:320, and anti-double-stranded DNA (dsDNA) 42 IU/ml. Chest CT showed multiple lymph node enlargement and calcification in mediastinum and bilateral hilum, multiple bullae in the upper lobe of both lungs, bronchial mucus plug formation and swelling incomplete in bilateral lower lobes, pleural effusion in bilateral pleural and bilateral pleura slightly thickened. The patient was diagnosed as ANCA-associated vasculitis. ANCA-associated vasculitis (AAV) is a group of autoimmune diseases that involve inflammation and necrosis of small blood vessels, including microscopic polyangiitis, granulomatosis with polyangiitis (formerly known as Wegener’s granulomatosis), and eosinophilic granulomatosis with polyangiitis (formerly known as Churg-Strauss syndrome). These diseases can cause damage to multiple organs and tissues.The production of ANCA, which are autoantibodies targeting neutrophil cytoplasmic components such as PR3 and MPO [4].

**Fig. 1:**
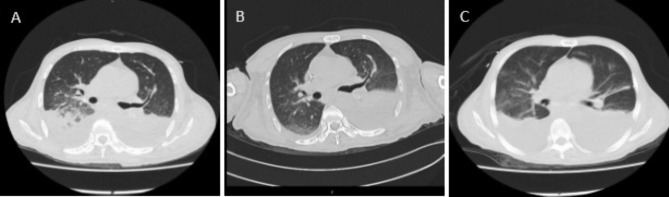
The chest computed tomography imaging during disease progression. Compared with the chest computed tomography on admission day and Day 10 (A and B), the inflammation on Day 17 progressed significantly (C)

**Fig. 2:**
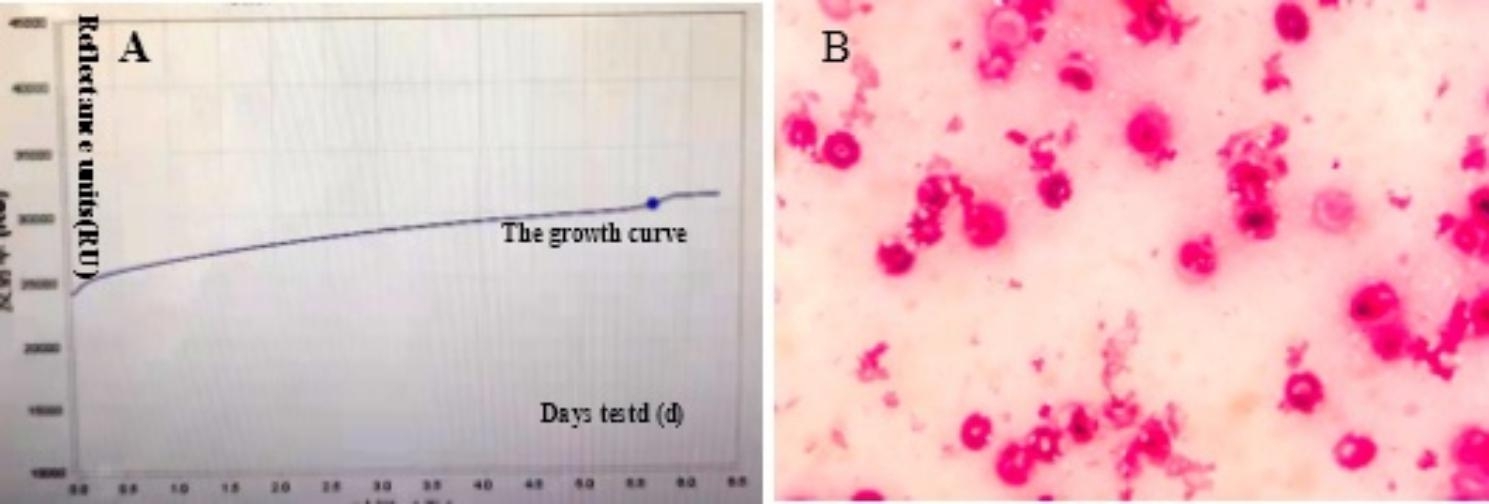
(A) Anaerobic bottles of blood culture reported positive after 135 h, while the growth curves were flat. (B) No bacteria were found in Gram staining under microscope

On day 16 after admission, he developed hyperthermia (38 ℃) and left epistaxis. Laboratory examinations revealed leukocytosis (5.22 × 10^9^/L, neutrophils 98.8%) with high serum C-reactive protein (44.21 mg/L,normal range 0–10 mg/L) and high procalcitonin level (2.25 ng/mL, normal range 0-0.5 ng/L). Haemoglobin (49 g/L, normal range 120–160 g/L)and platelet count (48 × 10^9^/L, normal range 100-300 × 10^9^/L) was decreased. Elevate IL-6 level was 21.43 pg/mL (normal range 0–5.3 pg/mL). Chest CT showed right bilateral pulmonary inflammation with significantly increased pleural effusion progression (Fig. 1). Blood cultures were obtained then and empirical antibiotics treatment with voriconazole (350 m g iv.drip Q12h), sulbactam (1 g iv Q8h) and imipenem (1000 m g iv.drip Q8h). Subsequently, both sets of anaerobic bottles for blood culture reported positive after 135 h, but the growth curves appeared flat. After Gram staining of blood smears, no bacteria were found (Fig. 2). After 24 h of incubation, no bacterial growth was found on the blood agar plate, and it was initially suspected to be false positive. However, after the next 7 days of antibiotics treatment, the patient’s body temperature did not decrease significantly. Antibiotics were adjusted to polymyxin B (1 million u iv.drip QD) and cefoperazone-sulbactam (1.5 g iv.drip Q12h).

**Fig. 3:**
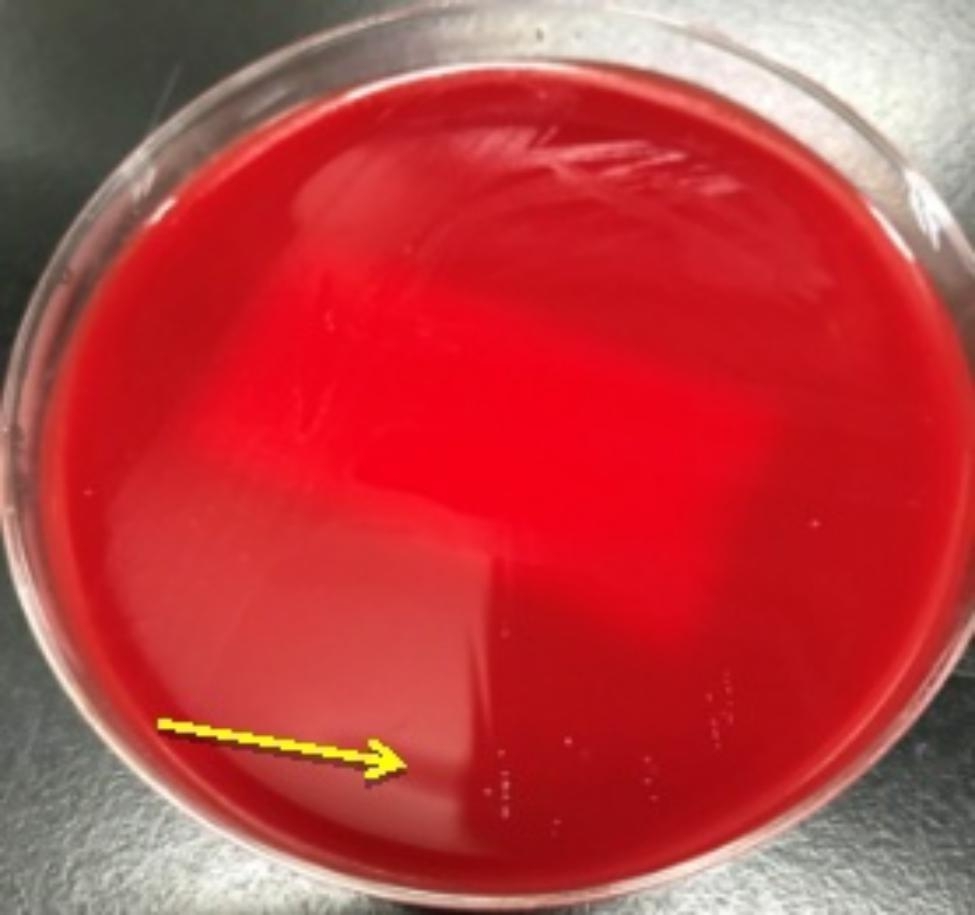
Needle-tip colonies appeared on the blood-agar plate 48 h after incubation

**Fig. 4:**
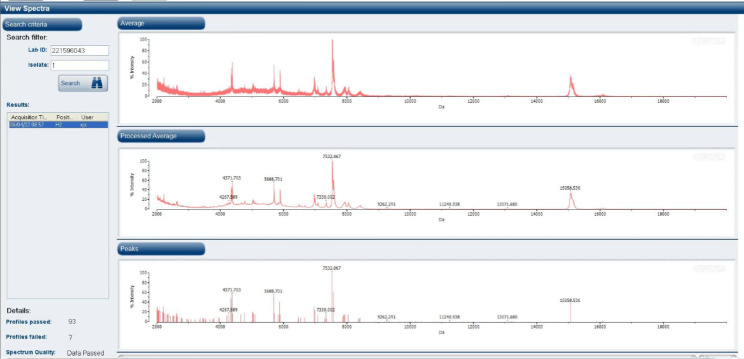
It was identified as *Mycoplasma hominis* by matrix-assisted laser desorption ionization time-of-flight mass spectrometry

**Fig. 5:**
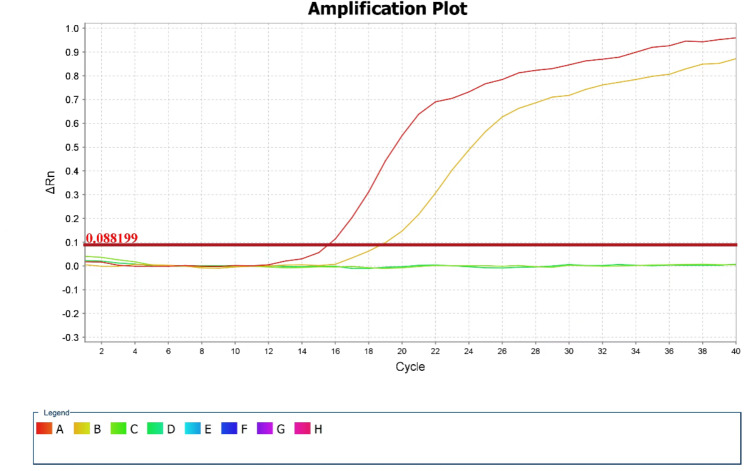
qPCR analysis of the Mycoplasma hominiss. We have detected blood of the patient by quantitative polymerase chain reaction with specific primers (Forward primer: CCGTTCAAGCTACCCGAACA, reverse primer: AATGCAAGCCCTCAAGGAAA) for *Mycoplasma hominis* we designed, which indicated infection of *Mycoplasma hominis*. (The yellow and red line represented two replicate for blood of the patient. The green lines represented two replicate for negative template control.)

On Day 30, the patient suffered from epistaxis again. He displayed recurrent fever with the maximum temperature 38.3℃. Blood cultures were obtained again and metagenomic next generation sequencing (mNGS) for the peripheral blood specimen was performed. After sample processing and DNA extraction [QIAamp® UCP Pathogen DNA Kit (Qiagen)] for mNGS, libraries were constructed for the DNA samples using a Nextera XT DNA Library Prep Kit (Illumina, San Diego, America), sequencing was performed using Nextseq 550Dx sequencer (Illumina, San Diego, America). The rules of bioinformatic analysis for mNGS result and threshold criteria for interpretation of metagenomic analysis were referred to our previous process [5]. Within 48 h, the mNGS detected 19 total reads corresponding to *Mycoplasma hominis*, with 0.2% coverage and 4.6% relative abundance in bacteria (Table 1). Specifically, the total sequencing output was 32,513,857 reads, and 31,079,967 of them were valid sequences for analysis, with the host rate 98.13%. The accession number used for mapping of the reference genome is NZ_JRWZ01000004. The 0.2% mapping was covered by the sequence-specific to *M. hominis* and the confidence level was 99%. Meanwhile, both sets of anaerobic bottles for blood culture reported positive after 125.9 h, but the growth curves were still flat. After Gram staining of blood smears, no bacteria were found also. Based on the results of mNGS, we suspected that the pathogen of bloodstream infection was most likely *Mycoplasma hominis*, since it lacked cell wall and no bacteria was found under Gram staining. Therefore, we deliberately extended the incubation time of the strain. 48 h after incubation, needle—like colonies appeared on the blood-agar plate (Fig. 3). It was identified as *Mycoplasma hominis* by matrix-assisted laser desorption ionization time-of-flight mass spectrometry (MALDI-TOF MS) (bio-Merieux, Durham, NC) (Fig. 4). To confirm the diagnosis, we performed qPCR analysis and *Mycoplasma hominis* was further confirmed (Fig. 5).

**Fig. 6:**
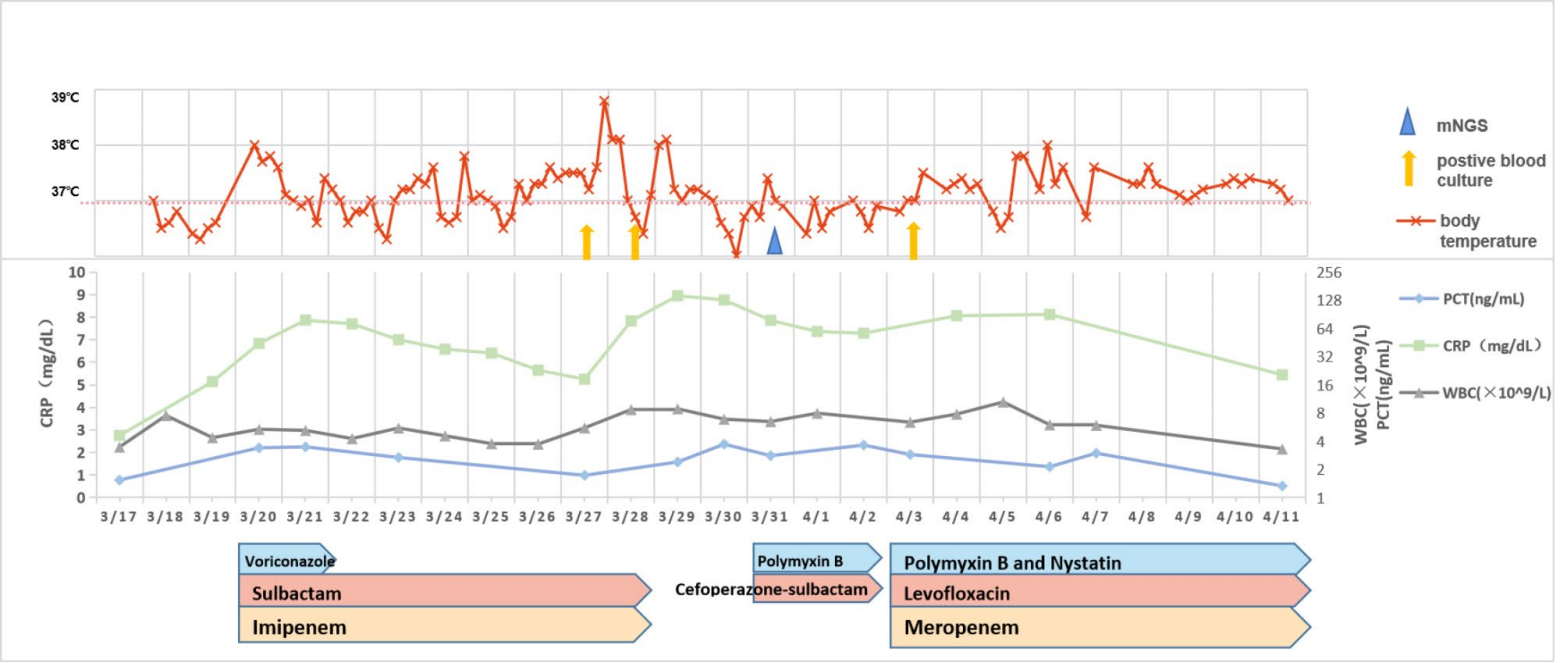
Treatment timeline including changes of CRP, PCT, WBC, and etiology detection timepoints. (CRP, c-reactive protein; PCT, procalcitonin; WBC, white blood cell)

Based on these results, combination therapy with levofloxacin (500 mg P.O QD), polymyxin B (500 million u iv.drip Q12h), and meropenem (0.5 g iv Q8h) were used (Fig. 6). After treatment for 9 days, the patient’s infection was gradually controlled and body temperature returned to normal. The patient was discharged without further complications. During the three-month outpatient follow-up, no recurrence of symptoms or signs was reported.

**Table 1:** The micro-organisms were detected by NGS

Category	Pathogens	Reads	Coverage (%)	Relative abundance (%)
Bacteria	*Mycoplasma huminis*	19	0.2	4.6
Fungi	-	-	-	-
Virus	-	-	-	-
Parasite	-	-	-	-
Human microecological microflora	*Corynebacterium imitans* *Pseudomonas oleovorans* *Staphylococcus epidermidis* *Staphylococcus capitis* *Micrococcus luteus* *Others*	15 12 9 2 11 -	0.02 0.04 0.03 - 0.06 -	1.9 1.8 0.94 0.94 0.78 89.04

## Discussion

*Mycoplasma hominis* has no cell wall and is highly polymorphic. It is the only pathogenic *Mycoplasma* that can grow on common bacterial media such as blood and chocolate media. *M. hominis* mainly resides in the genitourinary tract and respiratory tract [6], and also causes neonatal infection [7, 8], systemic infection [9] and immunosuppression [10]. In recent years, sporadic infections caused by *M. hominis* have been reported. Stijn et al. reported a surgical patient with bloodstream infection and pneumonia caused by *M. hominis*, whose prostatic abscess was identified as a possible major source of infection [11]. The key to successful treatment was due to the abscess drainage and treatment with doxycycline. Allan et al. reported four cases of *M. hominis* infections in 3 heart and 1 bilateral sequential lung transplant recipients hospitalized for more than 3 weeks. Treatment was successful through early active surgical intervention and treatment with a combination of antibiotics comprisingclindamycin, doxycycline and/or ciprofloxacin [12].

However, *M.hominis* is diffcult to diagnose due to its elusiveness and fastidious slow-growing nature. This may be explained for the followingreasons. First, *M. hominis* is surrounded by a three layered of cell membrane, but lacks a cell wall [13]. Therefore, Gram staining cannot penetrate [14, 15], which makes it difficult to identify clinically. Second, it usually takes several days (often ≥ 2 days) to grow into tiny colonies on the conventional culture medium,, which reduces the possibility of timely diagnosis [16]. Therefore, an early and rapid diagnostic method is urgently needed in clinic.

This case involved ANCA associated vasculitis complicated with sepsis. When the patient’s first set of blood cultures reported positive by the BACT/ALERT® VIRTUO® blood culture detection system (bioMérieux, France), no pathogenic organism was found under Gram staining. Furthermore, no colonies were found on the plate 24 h after the blood sample was transferred to a blood agar plate,. Combined with the slow growth curve and the positive reporting time of more than 120 h, the blood culture was initially suspected to be false positive. After receiving the empirical anti-infection treatment of meropenem for 3 days, the anti-infection effect was not obvious. Subsequently, NGS of peripheral blood showed that the patient was infected with *M. hominis*. Based on this result, we extended the incubation time of the blood agar plate of the second set of positive blood culture. After 48 h, we observed needle-tip colony growth on the plate. It was identified as *M. hominis* by MALDI-TOF (with identity of 99.9%). In this case, we found that the positive blood culture caused by *M. hominis* was easily missed. Therefore, if small needle like colonies grow on the blood plate, but no pathogen is found by Gram staining, the possibility of *Mycoplasma spp.* should be considered. The positive blood culture bottles in this case were all anaerobic bottles, which was consistent with other reported cases [17]. We speculated that the addition of polyaniline sulfonate and anticoagulant in the aerobic bottle may inhibit the growth of *Mycoplasma.*

NGS has the advantages of unbiased sequencing by extracting total DNA or RNA, library preparation and deep sequencing from original samples [18]. It displays a rapid, comprehensive and highly sensitive performance in etiological diagnosis of difficult and severe cases [19, 20]. In this case, under the guidance of NGS, we deliberately extended the incubation time of blood culture medium transferred to blood agar plate. Moreover, MALDI-TOF MS shows an excellent performance for the identification of *M. hominis* [21, 22].

For the inference of the primary infection foci of the patients infected with *M. hominis*, the clinician ruled out the source of the reproductive and urinary tract by asking for medical history and physical examination. We believed that this patient with ANCA-associated vasculitis may have been caused by the colonization of thismycoplasma in the respiratory tract, which may have led to sepsis after vascular injury. However, the literature suggests that the transmission of this bacterium from the respiratory tract to the blood stream is very rare [23].

*M.hominis* is naturally resistant to all β-lactams because of the lack of a cell wall. Two antibiotic families, tetracyclines and fluoroquinolones showed strong action against these bacteria [24, 25]. More than 80% of *M. hominis* isolates were resistant to erythromycin, roxithromycin, azithromycin and clarithromycin. Josamycin, doxycycline and minocycline were most effective against *U. urealyticum* and *M. hominis* [26]. As a result, the antibiotic sensitivity profile of *M. hominis* can be utilized as a reference for the treatment of M. hominis BSI.

Briefly, *M. hominis* is difficult to culture and identify with ordinary methods. The application of NGS helps clinicians to make a rapid and precise diagnosis of *Mycoplasma* infection [27] in the near future. Further studies are required to improve the awareness of this mycoplasma and to develop effective therapies for patients with *M. hominis* sepsis.

## Data Availability

Data and materials of this report are publicly available. We uploaded the metagenome data of the peripheral blood specimenfrom a patient Metagenome. Pathogen reads were deposited in the Genome Warehouse in the National Genomics Data Center (National Genomics Data Center Members and Partners, 2023) uder project PRJNA943575, which are publicly accessible at http://www.ncbi.nlm.nih.gov/bioproject/943575.

## Abbreviations

ANCA: Antineutrophil cytoplasmic antibody
mNGS: metagenomic next generation sequencing
M hominis: Mycoplasma hominis
MALDI-TOF MS: Matrix-assisted laser desorption ionization time-of-flight mass spectrometry
dsDNA: Anti-double-stranded Deoxyribonucleic acid
p-ANCA: Perinuclear anti-neutrophil cytoplasmic
MPO: Myeloperoxidase
CT: Computed Tomography
iv: Intravenous injection
QD: Quaque die
Q12h: Every 12 h
Q8h: Every 8 h
P.O: peros
qPCR: Real-time Quantitative polymerase chain reaction
CRP: c-reactive protein
PCT: Procalcitonin
WBC: White blood cell
U.: urealyticum: Ureaplasma urealyticum
BSI: Bloodstream Infection
